# Mental health problems in children and adolescents in Germany. Results of the cross-sectional KiGGS Wave 2 study and trends

**DOI:** 10.17886/RKI-GBE-2018-084

**Published:** 2018-09-19

**Authors:** Kathrin Klipker, Franz Baumgarten, Kristin Göbel, Thomas Lampert, Heike Hölling

**Affiliations:** Robert Koch Institute, Berlin, Department of Epidemiology and Health Monitoring

**Keywords:** MENTAL HEALTH PROBLEMS, CHILDREN AND ADOLESCENTS, PREVALENCE AND TRENDS, HEALTH MONITORING, KIGGS

## Abstract

Mental health problems in children and adolescents are associated with individual and family-related constraints as well as social costs. 20.0% of children and adolescents showed mental health problems at the KiGGS baseline study (2003-2006). This study investigates the current prevalence for KiGGS Wave 2 (2014-2017) as well as time trends in comparison with the KiGGS baseline study. Mental health problems were assessed for 3- to 17-year-old children and adolescents by using the parent-based version of the Strengths and Difficulties Questionnaire (SDQ). For KiGGS Wave 2, the prevalence of mental health problems was 16.9%. A decreasing trend is pronounced particularly among boys between 9 and 17 years of age. Mental health problems are displayed more frequently by girls and boys from families with a low socioeconomic status compared to their peers from families with a medium or high socioeconomic status. These findings are discussed in the light of various measures and actions in health promotion and health care.

## Introduction

Mental health is a crucial prerequisite for a high quality of life, functional capacity and social participation. Emotional and behavioural problems in childhood and adolescence are often related to psychosocial impairments and can persist into adulthood. Specific psychological disorders in childhood and adolescence are associated with significant health-care costs [1, 2]. Considering the individual and societal consequences, it is important to investigate prevalence and time trends of mental health problems and psychological disorders in children and adolescents. Hence, appropriate measures and actions aiming at prevention and intervention can be initiated and evaluated. Based on representative data, the German Health Interview and Examination Survey for children and Adolescents (KiGGS) indicated that one in five children (i.e. 20.0%) showed mental health problems. Overall, boys were more likely to be affected by emotional and behavioural problems than girls, and children and adolescents from families with a low socioeconomic status showed poorer mental health than their peers from families with a higher socioeconomic status [3]. No difference was found in the proportion of children and adolescents that displayed mental health problems between the KiGGS baseline study (2003-2006) and the telephone survey in KiGGS Wave 1 (2009-2012) [3].

In light of the high and constant prevalence of mental health problems in childhood and adolescence, numerous measures for health promotion and health care have been initiated in recent years. These include the German government’s strategy to promote children’s health, which was adopted in 2008, the national health target ‘Growing up healthy’, the National Action Plan for a Germany Fit for Children 2005-2010, the establishment of the National Centre on Early Prevention in 2007, the 2012 law on Cooperation and Information in Child Protection, as well as the expansion of the medical check-ups for children (‘U’ examinations) in 2006. In addition, there has been a steady increase in the contractual medical care and in the utilization of psychiatric-psychotherapeutic services, especially among children and adolescents [4, 5].

**KiGGS Wave 2**

Second follow-up to the German Health Interview and Examination Survey for Children and Adolescents

**Data owner:** Robert Koch Institute

**Aim:** Providing reliable information on health status, health-related behaviour, living conditions, protective and risk factors, and health care among children, adolescents and young adults living in Germany, with the possibility of trend and longitudinal analyses

**Study design:** Combined cross-sectional and cohort study

**Cross-sectional study in KiGGS Wave 2**

**Age range:** 0-17 years

**Population:** Children and adolescents with permanent residence in Germany

**Sampling:** Samples from official residency registries - randomly selected children and adolescents from the 167 cities and municipalities covered by the KiGGS baseline study

**Sample size:** 15,023 participants

**KiGGS cohort study in KiGGS Wave 2**

**Age range:** 10-31 years

**Sampling:** Re-invitation of everyone who took part in the KiGGS baseline study and who was willing to participate in a follow-up

**Sample size:** 10,853 participants

**KiGGS survey waves**

► KiGGS baseline study (2003-2006), examination and interview survey
► KiGGS Wave 1 (2009-2012), interview survey
► KiGGS Wave 2 (2014-2017), examination and interview survey

More information is available at www.kiggs-studie.de/english

Based on representative cross-sectional data from the second wave of the KiGGS study (2014-2017), this article investigates the current prevalence of mental health problems in childhood and adolescence in Germany.

Furthermore, it describes time trends of mental health problems among children and adolescents using the KiGGS baseline study (2003-2006).

## Indicator

The German Health Interview and Examination Survey for Children and Adolescents (KiGGS) is part of the health monitoring system established at the Robert Koch Institute. KiGGS includes repeated cross-sectional surveys of children and adolescents aged between 0 and 17 years (KiGGS cross-sectional study). Both the KiGGS baseline study (2003-2006) and KiGGS Wave 2 (2014-2017) were conducted as a combined examination and interview survey. A detailed description of the methodology used in KiGGS Wave 2 can be found in New data for action. Data collection for KiGGS Wave 2 has been completed in issue S3/2017 as well as in KiGGS Wave 2 cross-sectional study – participant acquisition, response rates and representativeness in issue 1/2018 of the Journal of Health Monitoring [6, 7].

Mental health problems were assessed for children and adolescents aged 3 to 17 years using the parent-based version of the Strengths and Difficulties Questionnaire (SDQ) for the KiGGS baseline study and KiGGS Wave 2. Four problem areas of the SDQ were used to assess mental health: emotional problems, peer problems, behaviour problems and hyperactivity. Parents rated a total of 20 statements about their children as either ‘not true’ (0), ‘somewhat true’ (1) or ‘certainly true’ (2). Participants with a sum score of 12 points or less were categorized as children and adolescents without mental health problems, whereas those with 13 points or more were categorized as children and adolescents with mental health problems.

The analyses are based on data collected from 14,477 children and adolescents surveyed for the KiGGS baseline study (7,100 girls, 7,377 boys) and 13,205 children and adolescents who participated in KiGGS Wave 2 (6,637 girls, 6,568 boys). All participants were between 3 and 17 years of age. The results report prevalences stratified by gender, age and socioeconomic status (SES, [10]) with 95% confidence intervals (95% CI). The calculations were carried out using a weighting factor that corrects for deviations within the sample from the German population with regard to age in years, gender, federal state, nationality and the parents’ level of education (Mikrozensus, 2013 [11]). The calculation of p-values for time trends between the KiGGS waves was performed on the basis of age-standardised prevalences (population on 31 December 2015). Differences were examined using univariate logistic regression. A statistically significant difference between groups was assumed to have been demonstrated in cases where p-values were lower than 0.05.

## Results and discussion

**Figure 1:**
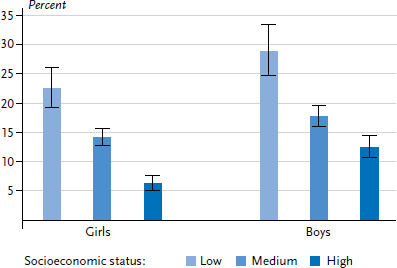
Prevalence of mental health problems according to gender and socioeconomic status (n=6,637 girls, n=6,568 boys) Source: KiGGS Wave 2 (2014-2017)

Overall, 16.9% of children and adolescents in Germany show mental health problems between 2014 and 2017 (Table 1). A significantly higher prevalence is found among boys than girls (19.1% versus 14.5%). This is particular true for children and adolescents between 3 and 14 years of age. No gender differences are found in the proportion of mental health problems for girls and boys aged between 15 and 17 years. The prevalence of children and adolescents with mental health problems is significantly higher in families with a low SES compared to their peers from families with a higher SES (Figure 1). Nearly one in four girls and almost one in three boys from families with a low socioeconomic status show emotional and behavioural problems, whereas only about one in fifteen girls and one in eight boys from families with a high socioeconomic status show poor mental health. One in seven girls and one in six boys from families with a medium socioeconomic status reported mental health problems. Overall, the difference between the frequency of mental health problems identified among children and adolescents from families with a high socioeconomic status (9.7%) and those with a medium status (16.1%) is lower than the difference between the frequency identified among children and adolescents from families with a medium socioeconomic status and their peers from families with a low socioeconomic status (26.0%).

The current findings from KiGGS Wave 2 confirm the patterns observed in the KiGGS baseline study with regard to the differences in mental health in terms of gender, age and socioeconomic status among children and adolescents living in Germany [12].

Compared to the KiGGS baseline study (2003-2006), the results of KiGGS Wave 2 (2014-2017) indicate a significant decrease in mental health problems (from 19.9% to 16.9%, Table 1). In comparison to the KiGGS publication in 2007 [12], the minimal difference in the indicated prevalence at KiGGS baseline is due to the adjusted weighting procedure (age-standardised prevalence according to the population on 31 December 2015) necessary for the calculation of time trends. A detailed analysis for girls and boys within different age groups reveals a statistically significant decrease for mental health problems among boys between 9 and 17 years of age. In contrast to the KiGGS baseline study, the data collected for KiGGS Wave 2 demonstrates that only about one in six boys (instead of one in four) currently displays emotional and behavioural problems. However, there is no comparable statistically significant decline in the frequency of mental health problems among girls across all age groups (Table 1). At KiGGS Wave 2, the known gender gap for mental health problems reported for the KiGGS baseline study (girls 15.9%, boys 23.6%) [12] seems to be closing due to the decrease in mental health problems among boys (girls 14.9%, boys 19.1%). Findings of either constant or reduced mental health problems among children and adolescents are consistent with current results from international studies [13-15].

One of the reasons for the decline in parent-reported mental health problems among children and adolescents living in Germany at KiGGS Wave 2 could be the implementation of health policy measures in the fields of health promotion and health care. During the period of the KiGGS baseline study, about 70% of children and adolescents who showed mental health problems did not seek psychiatric or psychotherapeutic treatment [16, 17]. Thus, the level of psychiatric-psychotherapeutic care provision appeared to be inadequate during the time of the KiGGS baseline study. Since then, the number of child and adolescent psychiatrists registered in the statutory health insurance has almost doubled (from 557 in 2003 to 1,062 in 2017) [5]. Consequently, an improved health care provision could have contributed to the decline in mental health problems for children and adolescents at KiGGS Wave 2.

In addition, measures to prevent psychological disorders and promote mental health were initiated. Alongside numerous projects in nurseries and schools, the extension of the regular child screening examinations may have contributed to a better prevention of psychological disorders. Since 2006, the so called “U10” and “U11” medical checkups for children between 7 and 10 years of age include a specific assessment of behavioural problems. Problems that are identified during check-ups can be discussed with the physician. Moreover, patients are provided with relevant information or support regarding the prevention of mental health problems in adolescence and adulthood. The implementation of medical check-ups (U10/U11) may explain the time trend results showing a decline in the prevalence of mental health problems in particular among boys at the age of 9 years.

Furthermore, economic and political changes may be associated with the decline in parent-reported emotional and behavioural problems among children and adolescents. For example, since the KiGGS baseline study, important family policy measures have been implemented in child day-care (i.e. parental benefits, day-care provision and tax advantages) contributing to a significant increase in the use of day-care facilities outside of the family home between 2007 and 2013 [18]. Therefore, it seems plausible in the context of growing professional demands and increasing numbers of working mothers that these developments in child day-care may have reduced some of the burden associated with family life [19].

However, the current data cannot answer the question conclusively to which extent these and other societal changes (e.g. economic stability and lower unemployment rates) have contributed to the decline of parent-reported mental health problems between the KiGGS baseline study and KiGGS Wave 2 among children and adolescents living in Germany.

The time trend between the KiGGS baseline study and KiGGS Wave 2 reveals a significant decrease in parent-reported mental health problems among boys, but not among girls. However, emotional and behavioural problems are particularly evident when the symptoms are more visible and perceptible, for example in the form of oppositional or hyperactive behaviour. In contrast, mental health problems categorized by rather inward symptoms, such as sadness and social withdrawal, are less obvious. Therefore, one possible explanation for gender differences could be that health care measures are effective for predominantly apparent symptoms that have a negative impact on activities in nurseries, schools or the family. Since boys are affected more frequently by visible and perceptible mental health problems than girls [12], parents and social environment might notice the need for action more quickly. It is plausible that earlier intervention in oppositional or hyperactive behaviour has a preventive effect on further comorbid symptoms (such as peer relationship problems), which in turn could have contributed to the decline in mental health problems among boys.

Although the study identified a decline in mental health problems, the frequency of emotional and behavioural problems among children and adolescents still remains at a high level. It should be noted, that the SDQ as an indicator of emotional and behavioural problems does not cover the entire breadth of mental health in childhood and adolescence. As such, the SDQ does not enable valid conclusions about specific psychological disorders or whether mental health problems require treatment. However, in light of the reduced quality of life and increased medical costs linked to children and adolescents with mental health problems [2, 20], it is crucial that young people and their families are informed about the opportunities available for resource-enhancing prevention and intervention, as well as the appropriate forms of medical and psychosocial care. This applies particularly to children and adolescents from families with a low socioeconomic status, as they continue to show poor mental health more frequently than their peers. Finally, the health care system is faced with the enormous task to recognise the various forms of mental health problems as early as possible and to accommodate better to mental health problems with symptoms that are less visible and more common in girls than in boys.

## Key statements

- According to information provided by their parents, 16.9% of children and adolescents living in Germany displayed mental health problems at KiGGS Wave 2 (2014-2017).
- In comparison to the KiGGS baseline study (2003-2006), the proportion of children and adolescents with parent-reported mental health problems is reduced by about three percentage points.
- The proportion of children and adolescents with mental health problems has dropped predominantly among boys between 9 and 17 years.
- Boys are still affected more frequently by mental health problems than girls at KiGGS Wave 2 (2014-2017).
- Children and adolescents from families with a low socioeconomic status are more than twice as likely to show mental health problems as their peers from families with a high socioeconomic status.

## Figures and Tables

**Table 1:** Prevalence of mental health problems according to gender and age at the KiGGS baseline study (n=7,100 girls, n=7,377 boys) and KiGGS Wave 2 (n=6,637 girls, n=6,568 boys) Source: KiGGS baseline study (2003-2006), KiGGS Wave 2 (2014-2017)

	KiGGS baseline study		KiGGS Wave 2	
	%	(95% CI)	%	(95% CI)
**Girls**	**15.9**	**(14.9-17.0)**	**14.5**	**(13.2-15.9)**
**Age group**				
3-5 Years	17.2	(14.7-19.9)	13.9	(11.2-17.1)
6-8 Years	14.7	(12.4-17.4)	13.8	(11.6-16.2)
9-11 Years	18.6	(16.5-21.0)	16.4	(13.3-20.1)
12-14 Years	15.9	(13.8-18.3)	13.9	(11.9-16.3)
15-17 Years	13.4	(11.5-15.6)	14.6	(12.2-17.3)
**Boys**	**23.6**	**(22.3-24.9)**	**19.1**	**(17.7-20.6)**
**Age group**				
3-5 Years	21.4	(18.9-24.2)	20.9	(17.5-24.7)
6-8 Years	25.3	(22.7-28.2)	22.3	(19.4-25.4)
9-11 Years	28.8	(26.2-31.7)	22.2	(19.0-25.7)
12-14 Years	25.8	(23.1-28.9)	19.2	(16.6-22.0)
15-17 Years	17.2	(14.8-20.0)	12.2	(9.9-15.0)
**Total (girls and boys)**	**19.9**	**(19.0-20.8)**	**16.9**	**(15.9-17.9)**

CI=confidence interval
